# Polymeric Matrix Mini-Tablets Based on Eudragit^®^ S 100 and HPMC for Controlled Release of Pantoprazole

**DOI:** 10.3390/pharmaceutics18030327

**Published:** 2026-03-05

**Authors:** Hugo Pardo, Mª Ángeles Peña, Borja Martínez-Alonso, Carlos Torrado-Salmerón, Víctor Guarnizo-Herrero

**Affiliations:** 1 Department of Biomedical Sciences, Faculty of Pharmacy, University of Alcalá, Alcalá de Henares, 28871 Madrid, Spain; hugo.pardo@edu.uah.es (H.P.); angeles.pena@uah.es (M.Á.P.); borja.martineza@uah.es (B.M.-A.); 2 Department of Pharmaceutics and Food Technology, Faculty of Pharmacy, Complutense University of Madrid, Plaza Ramón y Cajal s/n, 28040 Madrid, Spain; ctorrado@ucm.es; 3 University Institute of Industrial Pharmacy (IUFI), Complutense University of Madrid, 28040 Madrid, Spain

**Keywords:** pantoprazole, drug release, kinetics, mini-tablets, PCA

## Abstract

**Background:** Pantoprazole is a widely used proton pump inhibitor that is highly unstable under acidic conditions. This limits the performance of conventional formulations and typically requires enteric-coated dosage forms or alternative modified-release approaches. This study reports the development of polymeric matrix mini-tablets designed to protect pantoprazole during gastric exposure and to enable pH-dependent release under intestinal conditions. The formulations combine Eudragit^®^ S 100, a pH-dependent polymer, with HPMC, a hydrophilic matrix former that modulates drug release through hydration and swelling. **Methods:** Matrix mini-tablets were prepared by blending pantoprazole with selected excipients at optimised proportions and compressing the blends by direct compression using an eccentric tablet press. Powder blends and mini-tablets were characterised according to pharmacopoeial specifications. Analytical techniques—including High-Performance Liquid Chromatography (HPLC), Differential Scanning Calorimetry (DSC), Fourier-Transform Infrared Absorption Spectroscopy (FT-IR), Powder X-Ray Diffraction (PXRD), and Scanning Electron Microscopy (SEM)—were employed to evaluate drug content uniformity, thermal behaviour, and potential drug–excipient interactions. In vitro dissolution studies were performed under sequential pH conditions, and the release kinetics were analysed using mathematical models. **Results:** Dissolution testing identified formulations F2 and F6 as providing the most suitable gastro-resistant performance in the acidic stage, together with sustained release up to 24 h. Kinetic modelling supported formulation-dependent release mechanisms, and multivariate analysis (PCA) highlighted relationships between physico-mechanical attributes and drug-release behaviour. **Conclusions:** The proposed matrix system shows potential as a robust, coating-free platform for the modified delivery of acid-labile drugs using direct compression, simplifying manufacturing. These findings support the rational design of oral modified-release formulations based on polymeric matrices.

## 1. Introduction

Pantoprazole is a proton pump inhibitor that is highly susceptible to degradation under acidic conditions. Therefore, formulation strategies focus on protecting the drug in the stomach and ensuring its release under the near-neutral pH conditions of the small intestine [1,2].

The oral route remains the most widely used approach for drug administration due to its convenience and high patient acceptance. Solid dosage forms are particularly preferred because they are easy to administer and support self-medication, thereby improving adherence. Among solid manufacturing methods, direct compression is especially attractive owing to its simplicity, cost-effectiveness, and avoidance of organic solvents and complex processing steps that require special coating equipment [2,3]. However, conventional immediate-release formulations may lead to pronounced plasma concentration peaks of the active pharmaceutical ingredient (API) followed by sub-therapeutic levels. Controlled-release systems have, therefore, been developed to reduce dose fluctuations and improve therapeutic efficacy and safety [4,5]. This approach is advantageous for drugs sensitive to acid conditions (pH 1.2–2.5). Given their stability profile in basic media, it is essential to ensure controlled drug delivery to a more suitable target site, optimising therapeutic efficacy under specific environmental conditions [6]. Controlled drug delivery aims to achieve appropriate temporal and spatial drug distribution while reducing the dose and dosing frequency [5,7]. In this context, polymer-based matrix systems represent a practical strategy, as drug release can be modulated through the physico-chemical properties of the polymer(s), including hydration, swelling, and erosion behaviour. Instead of previous strategies, the integration of both a time-controlled and pH-dependent responsive mechanism allows for greater versatility in formulation delivery systems, facilitating the achievement of specific therapeutic goals, as demonstrated in the literature [3,8,9]. Consequently, systematic characterisation and optimisation of polymer composition are essential to ensure manufacturing reproducibility and predictable dosage form performance.

Drug-release behaviour is largely governed by the physico-chemical properties of the polymeric matrix; therefore, systematic characterisation and optimisation of these properties are essential to ensure reproducible manufacturing and predictable dosage form performance. In general, modified-release oral systems aim to (i) sustain drug release over a prolonged period, and (ii) reduce fluctuations in drug exposure by controlling the timing and, when applicable, the gastrointestinal site of release [4,5,10].

Drug delivery systems are commonly classified as reservoir systems or matrix devices. In matrix devices, the drug is homogeneously dispersed within a continuous phase, which may be hydrophilic or hydrophobic [3]. Matrix systems are generally cost-effective, easy to scale up, and can attenuate the variability in plasma drug concentrations [6,11]. Depending on the materials used, matrix systems have been described as hydrophilic, hydrophobic, lipid-based, plastic, or biodegradable [9,12]. In aqueous media, modified-release matrices undergo an initial hydration step, leading to the formation of a superficial gel layer. As the medium penetrates the matrix, the gel layer expands, and drug release proceeds through diffusion across the gel barrier and/or erosion of the matrix surface [2,4]. The resulting kinetics are governed by medium penetration and polymer relaxation processes (hydration and swelling), and are influenced by drug properties (e.g., solubility and molecular weight), formulation variables (e.g., geometry and manufacturing method), and polymer-related factors such as type, viscosity, and blend proportions [1].

By optimising polymer composition and matrix content, a wide range of release rates can be achieved by modulating drug diffusivity through the gelled structure. Several studies have reported the use of hydroxypropyl methylcellulose (HPMC), time-dependent, in combination with other polymers, including sodium alginate [10,13] and Eudragit^®^ S 100, pH-dependent ref. [7], to tailor release behaviour. Eudragit^®^ S 100 is an anionic copolymer of methacrylic acid and methyl methacrylate, a pH-dependent polymer designed to remain intact under gastric conditions and to dissolve at higher pH values in the lower gastrointestinal tract, at pH 7.0, enabling targeted release and improved stability for acid-labile drugs [1,4,10,11,13,14]. In turn, HPMC is widely used as a hydrophilic matrix, since high viscosity can help maintain tablet integrity and provide mechanical strength through gastrointestinal (GI) transit; upon hydration and swelling, it forms a viscous gel layer around the tablet surface, acting as a barrier to control drug diffusion and modulating drug diffusion and erosion over extended periods [1,14]. This approach combines two or more excipients that synergically enhance the functional properties without altering their chemical structure, which has been represented in different studies [8]. Such strategies address the inherent limitations of single-mechanism delivery systems; for instance, pH-dependent systems are often restricted by significant inter- and intra-individual variability in gastrointestinal pH and gastric emptying rates. Similarly, time-dependent release can fluctuate based on intestinal transit times [15]. Consequently, integrating a pH-dependent polymer with a hydrophilic matrix offers a robust, rational design to protect pantoprazole from gastric acidity while ensuring a controlled and sustained release profile. This dual-mechanism strategy has been successfully exemplified in various studies combining enteric and swellable polymers [6,8,15]. In summary, the synergy between pH-dependent and matrix-controlled diffusion minimises physiological interference, ensuring reliable therapeutic performance for acid-labile drugs like pantoprazole [16,17].

While traditional pantoprazole stabilisation relies on complex, costly enteric coating processes, this study develops an efficient alternative: polymeric matrix mini-tablets. By combining Eudragit S^®^100 and HPMC, a robust gastro-resistant system was achieved through a single-step direct-compression approach. This approach simplifies manufacturing, eliminates organic solvents, and leverages the mini-tablet format for improved intestinal transit and patient compliance. Future research will focus on in vivo studies to establish an in vitro–in vivo correlation (IVIVC), alongside a deeper characterisation of the polymeric matrix properties, such as swelling dynamics and mechanical integrity during erosion.

## 2. Materials and Methods

### 2.1. Raw Materials

Pantoprazole powder was supplied by ROVI laboratories (ROVI, Madrid, Spain); Eudragit^®^ S 100 was obtained from Evonik (Evonik industries, Darmstadt, Germany). HPMC was purchased from Jescuder S.L (Terrassa, Barcelona, Spain), and Compritol^®^ 888 ATO was supplied by Gattefossé (Gattefossé, Saint-Pries, France). For analytical procedures and dissolution media, hydrochloric acid (HCl), potassium dihydrogen phosphate (KH_2_PO_4_), and Sodium hydroxide (NaOH), all of analytical grade, were purchased from Panreac Química S.L.U. (Castellar de Vallès, Barcelona, Spain). HPLC-grade solvents such as methanol and acetonitrile were purchased from Merck KGaA (Darmstadt, Germany). Water was ultra-pure Milli-Q Merck KGaA (Darmstadt,, Germany). All other reagents and chemicals used were of laboratory analytical grade.

### 2.2. Blend Preparation and Pre-Compression Studies

Pantoprazole mini-tablets were prepared by direct compression. Pantoprazole, HPMC, Eudragit^®^ S 100, and Compritol^®^ 888 ATO were accurately weighed, as outlined in Table 1, and blended in a mortar until a homogeneous mixture was obtained. Pre-compression studies were conducted to assess the flowability and compressibility of the powder blends prior to tabletting and to support the selection of appropriate processing conditions. All parameters were determined according to the methodology and specifications of the *Real Farmacopea Española* (RFE).

#### 2.2.1. Bulk and Tapped Densities

Bulk density (Da) and tapped density (Dc) were determined following the method described in monograph 2.9.34 of the RFE [18]. A known mass of powder (m) was introduced into a graduated cylinder, and the unsettled volume (Vo) was recorded to calculate bulk density using PT-TD200 equipment (Pharmatest Services Ltd., Turku, Finland). The same sample was then mechanically tapped for 100 strokes, and the resulting volume (Vt) was recorded to calculate the tapped density. All measurements were performed in triplicate. Bulk density and tapped density were calculated using the following formulas:

(1) $$Da=\;\frac{m}{Vo}\;\;\;Dc=\;\frac{m}{Vt}$$

#### 2.2.2. Compressibility and Flowability

To further assess the compressibility and flowability of the powder blend, the compressibility index (Carr’s index, IC) and Hausner’s ratio (IH) were calculated from the bulk and tapped density values. IC determines the tendency and capacity of a powder blend to undergo compression, expressed as a percentage, whereas IH evaluates interparticle friction and packing ability as predictors of powder flowability, which is dimensionless. IC and IH values were subsequently classified according to the standardised criteria established in the Spanish Pharmacopoeia 2.9.36 of the RFE [19]. Both indices were calculated using the following formulas:

(2) $$IC=\;\left(\frac{Dc-Da}{Dc}\right)\;\times \;100\;\;\;IH=\;\frac{Dc}{Da}$$

#### 2.2.3. Angle of Repose (θ)

The angle of repose was determined according to RFE monograph 2.9.36 [19]. This parameter reflects powder flowability and cohesiveness by describing the stability of the heap formed under gravity. Briefly, a funnel was positioned vertically at a fixed height of 20 cm above a flat surface. In each experiment, 10 g of the powder blend was gently poured into the funnel without compaction. The funnel outlet was then opened to allow the powder to flow freely and form a conical heap. The angle of repose was calculated as follows:

(3) $$Tg(\theta )=\frac{h}{r}$$

where θ is the angle of repose expressed in degrees (°); h is the maximum pile height (cm); and r is the corrected mean radius (cm). The corrected radius was calculated by subtracting the diameter occupied by the funnel stem opening from the measured pile diameter.

### 2.3. Drug–Excipient Compatibility

#### 2.3.1. Differential Scanning Calorimetry (DSC)

DSC measurements were performed using a TA Instruments’ DSC (DSC 25, TA Instruments, New Castle, DE, USA). The instrument was temperature-calibrated using indium as the calibration reference standard. Samples (5 to 10 mg of mini-tablets and individual components) were weighed into hermetically sealed aluminium pans, with an empty pan used as reference. Analyses were conducted from 30 to 250 °C, at heating rates of 5 and 10 °C/min, under a nitrogen flow of 50 mL/min.

#### 2.3.2. Fourier-Transform Infrared Spectroscopy (FT-IR)

FT-IR spectra of pure pantoprazole, individual polymers, and formulation powders were recorded using a PerkinElmer Spectrum Two spectrophotometer (PerkinElmer, Waltham, MA, USA). Approximately 1 to 2 mg of each sample was analysed directly. Spectra were acquired by averaging 18 scans over the 4000 to 400 cm^−1^ range at a resolution of 1 cm^−1^. The characteristic absorption bands of pantoprazole were compared with those of the excipients and formulations to identify potential chemical interactions, including peak shifts or the appearance of new bands.

#### 2.3.3. Powder X-Ray Diffraction (PXRD)

PXRD patterns were recorded using a Philips X’Pert-MPD X-ray diffractometer (Malvern Panalytical, Almelo, The Netherlands) with Cu Kα radiation (λ = 1.54 Å), operated at 45 kV and 40 mA. Samples were mounted on a holder on a glass slide, gently compacted, and smoothed. Diffraction patterns were collected from 5° to 50° (2θ) using a step size of 0.04° and a counting time of 1 s per step.

#### 2.3.4. Scanning Electron Microscopy (SEM)

Samples were mounted on aluminium stubs and sputter-coated with a thin zirconium layer prior to analysis. SEM observations were performed using a JEOL IT1700 instrument (Tokyo, Japan). Surface morphology was examined using secondary electron imaging at an accelerating voltage of 5 kV and at magnifications of 2000×, 5000×, and 10,000×. Raw materials (pantoprazole, Eudragit^®^ S 100, and HPMC) and formulations F2, F6, and F9 were analysed.

### 2.4. Tablet Preparation

The final blend was then compressed using an eccentric tablet press (J. Bonal, Model B No. 508, Bonals Technologies Spain S.A., Barcelona, Spain) fitted with concave punches (set No. 6) to obtain mini-tablets with a target mass of 125 mg and a drug load of 20 mg per unit. All batches were stored in airtight, light-protected containers until further analysis.

### 2.5. Post-Compression Parameters

To evaluate the quality and mechanical integrity of the mini-tablets, post-compression tests were performed, including dimensional analysis, mass uniformity, hardness, friability, disintegration, content uniformity, and in vitro dissolution, in accordance with the RFE.

#### 2.5.1. Physical Characteristics

The thickness and diameter of 20 mini-tablets from each formulation were measured using a calibrated digital durometer (Pharmatest PTB 311E, Pharmatest Services Ltd., Turku, Finland). Results are reported as mean ± standard deviation.

#### 2.5.2. Mass Variation

Mass variation was assessed by individually weighing 20 mini-tablets from each formulation using an analytical balance (Mettler Toledo, Switzerland). The mean tablet mass and percentage deviation were calculated according to the acceptance criteria described in RFE chapter 2.9.5 [20].

#### 2.5.3. Hardness

Resistance to crushing (hardness) was evaluated according to RFE chapter 2.9.8 [21]. Ten units from each formulation were tested using a PTB 311E durometer (Pharmatest Services Ltd., Turku, Finland). Hardness values were recorded in newtons (N) for each unit, and the mean of 10 determinations was used as the representative value for each formulation.

#### 2.5.4. Friability

Friability (%) was determined using a Pharmatest PTF-E friability tester (Pharmatest Services Ltd., Turku, Finland) according to RFE chapter 2.9.7 [22]. A sample of mini-tablets equivalent to approximately 6.5 g was randomly selected and rotated for 100 revolutions at 25 rpm. After testing, the tablets were dedusted (W1) and reweighed (W2), and the percentage mass loss was calculated (Equation (4)). A mass loss greater than 1% was considered non-compliant with the pharmacopoeial limits.

(4) $$Friability\;\left(\%\right)=\frac{W1-W2}{W1}\;\times \;100$$

#### 2.5.5. Disintegration Time

Mini-tablet disintegration was determined according to RFE chapter 2.9.1 [23] using a PTZ-E disintegration tester (Pharmatest Services Ltd., Turku, Finland). Six mini-tablets were tested, placing one unit in each tube of the basket rack assembly. The test was performed in 900 mL of 0.1 M HCl maintained at 37 ± 0.5 °C, and the units were monitored for up to 2 h.

For prolonged-release dosage forms, the test is considered satisfactory when all units remain undisintegrated after 30 min, as described in the pharmacopoeial criteria [10].

#### 2.5.6. Content Uniformity Analysis

Content uniformity of the mini-tablets was evaluated in accordance with RFE chapter 2.9.6 [24]. Ten mini-tablets were individually weighed and powdered, and an accurately measured amount equivalent to one unit dose was transferred to a volumetric flask. The samples were dissolved, filtered through a 0.45 µm membrane, and the pantoprazole content was quantified by HPLC-UV using the validated method described below. Acceptance criteria were applied according to the pharmacopoeial limits for uniformity of content.

Chromatographic analysis was performed using an Agilent 1100 HPLC system equipped with a diode array detector (Agilent Technologies, Madrid, Spain). Separation was achieved on an ACE Excel C18 column (5 µm, 250 × 4.6 mm). The mobile phase consisted of an acetonitrile and aqueous phase (45:55 *v*/*v*), delivered at a flow rate of 1.0 mL/min, at ambient temperature. Detection was set at 285 nm, and the total run time was 5.4 min. Specificity was assessed by verifying the absence of interference at the retention time of pantoprazole.

Quantification was performed using a calibration curve prepared from pantoprazole sodium reference standard solutions in the range of 1.5 to 60 µg/mL. The analytical method was validated according to ICH Q2 (R2) guidelines [25]. Linearity across the tested range was assessed (R^2^ = 0.9998). Precision was evaluated by repeatability and intermediate precision, with RSD values ≤ 0.12%—within the pharmacopoeial limit (<2%). Specificity was assessed by the absence of interfering peaks from excipients or degradation products at the retention time of pantoprazole. Method sensitivity was established with a limit of detection (LOD) of 0.3 µg/mL and a limit of quantification (LOQ) of 0.5 µg/mL.

### 2.6. Analytical Method Validation and Drug–Polymer Compatibility

#### 2.6.1. Derivation of Drug Spectrum

A pantoprazole sodium solution (100 µg/mL) prepared in phosphate buffer (pH 6.8) was scanned to determine the maximum absorbance wavelength (lambda max). Calibration curves were constructed to assess linearity. In addition, the UV spectra of HPMC and Eudragit^®^ S 100 were recorded in the 200 to 400 nm range to evaluate potential spectral overlap at the selected wavelength for HPLC-UV quantification.

#### 2.6.2. In Vitro Dissolution (Drug-Release) Studies

In vitro dissolution studies were performed according to RFE chapter 2.9.3 [26], using a USP apparatus type II (paddle method). Each vessel contained 900 mL of dissolution medium maintained at 37 ± 0.5 °C. To simulate gastric-to-intestinal transit, sequential pH conditions were applied using media at pH 1.2 ± 0.2 and pH 6.8 ± 0.2. One mini-tablet was placed in each vessel, and the paddle rotation speed was set at 50 rpm.

Aliquots (6 mL) were withdrawn at predetermined time points (1, 2, 3, 4, 6, 7, 8, and 24 h) and replaced with an equal volume of fresh medium to maintain sink conditions. Samples were filtered, appropriately diluted when necessary, and analysed by HPLC-UV using the method described in Section 2.5.6. The cumulative percentage of pantoprazole released was calculated and plotted as a function of time to compare the release profiles among the developed formulations.

### 2.7. Release Kinetics Evaluation

Dissolution data were fitted to five kinetic models (zero-order, first-order, Higuchi, Korsmeyer–Peppas, and Hixson–Crowell) using linear regression of the corresponding linearised plots [27,28]. The goodness-of-fit was assessed using the coefficient of determination (R^2^). For the Korsmeyer–Peppas model, the release exponent (n) was obtained from the slope of the log(Mt/M infinity) versus log(t) plot [12,14]. The kinetic parameters (rate constants), n values, and R^2^ values are reported in Table 2.

## 3. Results and Discussion

In this section, the properties of the powder blends and the resulting mini-tablets are presented and discussed. Subsequently, the in vitro release behaviour is analysed, establishing the best-suited kinetic mechanism for each formulation. Finally, multivariate analysis is used to establish a quantitative relationship between the release properties and the physico-chemical characteristics of the formulations.

### 3.1. Pre-Compression Studies of the Powder Blend

All powder flow and packing properties were determined according to the procedures described in the RFE. Bulk and tapped densities were used to calculate compressibility indicators (Carr’s index and Hausner’s ratio), and the results are summarised in Table 3. Bulk and tapped density values ranged from 0.36 to 0.72 g/mL and from 0.47 to 0.96 g/mL, respectively, as seen in Table 3.

The angle of repose ranged from 13.3° to 26.97°. According to pharmacopoeial criteria, angles below 25° indicate satisfactory flow properties; accordingly, formulations F1, F7, and F9 exhibited excellent flow based on this parameter (θ < 20°), whereas formulation F6 was at the threshold of acceptability (≈27°).

**Figure 1:**
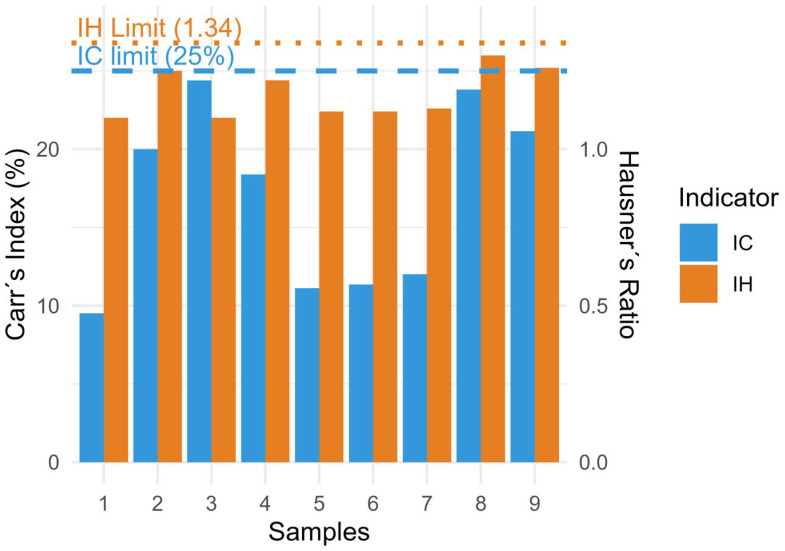
Comparative analysis of the flowability parameters across formulations. Blue bars represent Carr’s index (IC), and orange bars represent Hausner’s ratio (HR). The dashed lines represent the permissible limits for compression of the Carr’s Index (dashed line blue), and Hausner’s ratio (dashed line orange).

Carr’s index ranged from 9.52% to 24.39%. Formulations F1, F5, F6, and F7 showed values below 15%, indicating good compressibility behaviour, whereas formulations F3 and F8 exceeded 20%, suggesting limited flowability. However, Carr’s index does not reflect the speed and facility in compression process, which is reflected in [31]. Similarly, Hausner’s ratio values ranged from 1.10 to 1.32. Most blends exhibit acceptable flow, while F3 and F8 reach values of 1.31–1.32, indicating only moderate flow properties (Figure 1). This indicates that the particles are more cohesive, which may lead to poorer homogeneity during die filling in the compression process. In general, the majority of formulations exhibited satisfactory flowability and compressibility characteristics under direct-compression conditions, which is in accordance with the RFE. Nevertheless, the restricted flow evident for F3 and F8 has the potential to compromise process reproducibility and could influence die filling during the scale-up to high-speed compression, thereby affecting content uniformity. Consequently, the integration of suitable excipients to enhance flow may be necessary. 

### 3.2. Differential Scanning Calorimetry (DSC)

Differential Scanning Calorimetry (DSC) was performed to characterise the solid-state thermal behaviour of each component, including melting transitions, phase changes, and potential drug–excipient interactions.

**Figure 2:**
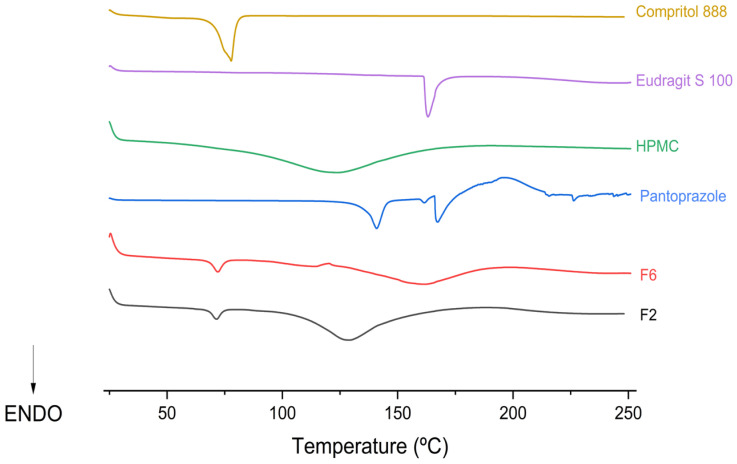
Thermograms of Compritol^®^ 888 ATO, Eudragit^®^ S 100, HPMC, and formulations F2 and F6.

The thermogram of sodium pantoprazole (Figure 2) exhibits an endothermic peak at 140.40 °C, corresponding to its melting point, in agreement with previously reported data [24]. Eudragit^®^ S 100 shows a pronounced endothermic event at 162.72 °C, consistent with its characteristic thermal transition. In contrast, HPMC displays a broader and less intense endothermic event around 119.79 °C, which is typically attributed to moisture loss and the amorphous nature of the polymer [1,26].

The thermogram of formulation F2, containing sodium pantoprazole, HPMC, Eudragit^®^ S 100, and Compritol^®^ 888 ATO, showed the absence of the characteristic melting peak of pantoprazole, suggesting an amorphous state and/or molecular dispersion within the polymeric matrix. The attenuation of polymer-related thermal events may indicate interactions among the components and overall formulation compatibility. Similarly, formulation F6 also lacked the characteristic pantoprazole melting peak, supporting the presence of an amorphous or molecularly dispersed drug within the matrix. The reduced intensity and slight shifts in polymer-related thermal events further suggest interactions among components and adequate compatibility within the formulation. Table 4 summarises the melting points of the different raw materials.

### 3.3. Fourier-Transform Infrared Absorption Spectroscopy (FT-IR)

**Figure 3:**
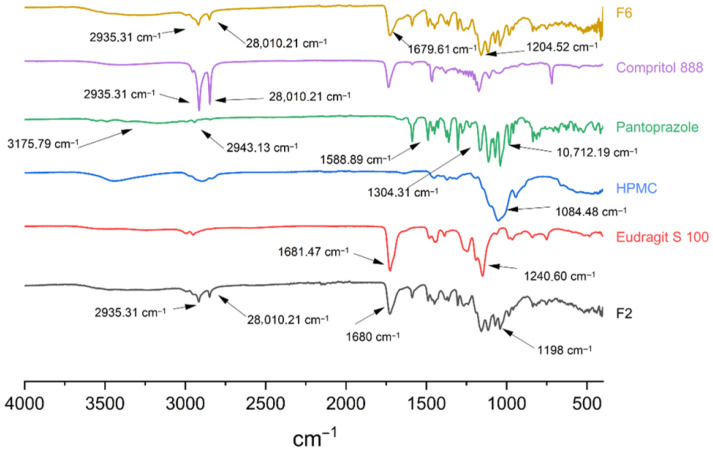
FT-IR spectra of Compritol^®^ 888, Eudragit^®^ S 100, HPMC and formulations F2 and F6.

The FT-IR spectra of the pure components and the formulated tablets are shown in Figure 3. In the spectrum of pantoprazole, the band at 3175.79 cm^−1^ is attributed to aromatic C–H stretching vibrations, while the band at 2943.13 cm^−1^ corresponds to aliphatic C–H stretching. The characteristic C=N stretching vibration is observed at 1588.89 cm^−1^. Additional bands at 1490.41, 1463.80, 1449.47, and 1427.29 cm^−1^ are associated with aromatic C=C stretching, consistent with the presence of aromatic rings. The sulfoxide (S=O) and CF_2_ functional groups, characteristic of pantoprazole, are identified by absorption bands at 1072.19 cm^−1^ and 1304.31 cm^−1^, respectively, while bands at 1040.78, 985.13, and 815.22 cm^−1^ are attributed to C–O and OCH_3_ stretching vibrations.

In the formulated tablets, the characteristic absorption bands of pantoprazole were preserved, indicating the absence of chemical interactions with the excipients. The broad O–H stretching band associated with HPMC and the ester-related bands of Eudragit^®^ S 100 confirm the presence of the polymeric matrix, whereas the aliphatic C–H stretching vibrations of Compritol^®^ 888 ATO are clearly observed in the 3000 to 2800 cm^−1^ region. Partial overlap and reduced intensity of pantoprazole bands in the 1250 to 1000 cm^−1^ region are attributed to spectral superposition rather than structural modification. Overall, the FT-IR results confirm the physico-chemical compatibility of pantoprazole with the excipients, in agreement with the previously reported literature [4].

For formulation F2, the FT-IR spectrum showed the characteristic bands of the individual excipients without evidence of chemical interactions among formulation components. The bands in the 3000 to 2800 cm^−1^ region were attributed to the aliphatic C–H stretching vibrations of Compritol^®^ 888 ATO. In addition, peaks at approximately 1680 cm^−1^ and 1198 cm^−1^ were clearly identified. The characteristic pantoprazole bands in the 1250 to 1000 cm^−1^ region appeared reduced or partially overlapped due to the broad absorption of HPMC, indicating physical superposition rather than chemical interaction.

### 3.4. Powder X-Ray Diffraction (PXRD)

A PXRD analysis was performed to investigate the solid-state characteristics of pantoprazole within the polymeric matrix tablets, and to assess formulation-induced changes in drug crystallinity across the different matrices. PXRD provides critical information on drug–polymer interactions, phase transformations, and halo phenomena, which may directly influence drug-release behaviour in hydrophilic and pH-dependent matrix systems.

**Figure 4:**
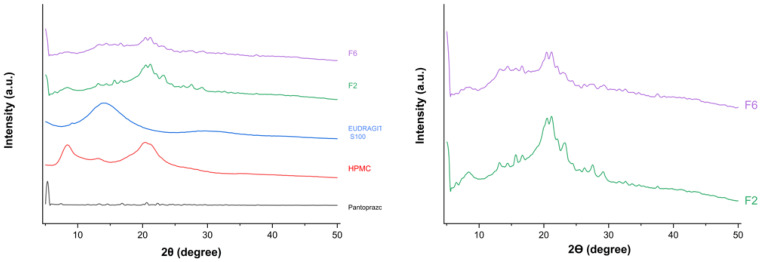
PXRD patterns of pure pantoprazole, Eudragit^®^ S 100, HPMC, and representative matrix formulations F2 and F6, including a magnified view of F2 and F6 patterns.

The diffractogram of pure pantoprazole (Figure 4) shows multiple sharp, intense reflections, confirming its crystalline nature. In contrast, HPMC and Eudragit^®^ S 100 exhibit low-intensity, diffuse patterns with a characteristic broad halo, consistent with predominantly amorphous structures. Characteristic reflections are observed for pantoprazole at approximately 5°, 10°, and 30° (2θ); for HPMC at approximately 8° and 20°; and for Eudragit^®^ S 100 at approximately 10° and 25°. Compared with the raw drug, all formulations (F1 to F9) display diffractograms dominated by the polymer background, with reduced intensity of the drug-related peaks, indicating a decrease in the apparent crystallinity of pantoprazole in the matrix systems.

Notably, formulation F2 shows greater attenuation of pantoprazole reflections than F6, suggesting a higher degree of amorphization and/or a more homogeneous dispersion of the drug within the polymeric matrix. This observation is consistent with the higher HPMC content in F2 compared with F6, and with the more pronounced drug encapsulation observed by SEM. These solid-state changes are in agreement with the DSC results and correlate with the dissolution behaviour, supporting the role of matrix composition in controlling drug release.

Overall, the PXRD findings confirm that polymer selection and matrix composition play a key role in modulating pantoprazole crystallinity and, consequently, the release performance of the modified-release matrix formulations.

### 3.5. Morphological Characterisation by Scanning Electron Microscopy (SEM)

Scanning Electron Microscopy (SEM) was used to characterise the raw materials and the surface morphology of the matrix formulations before and after processing, including pantoprazole, Eudragit^®^ S 100, HPMC, and formulations F1 to F9.

**Figure 5:**
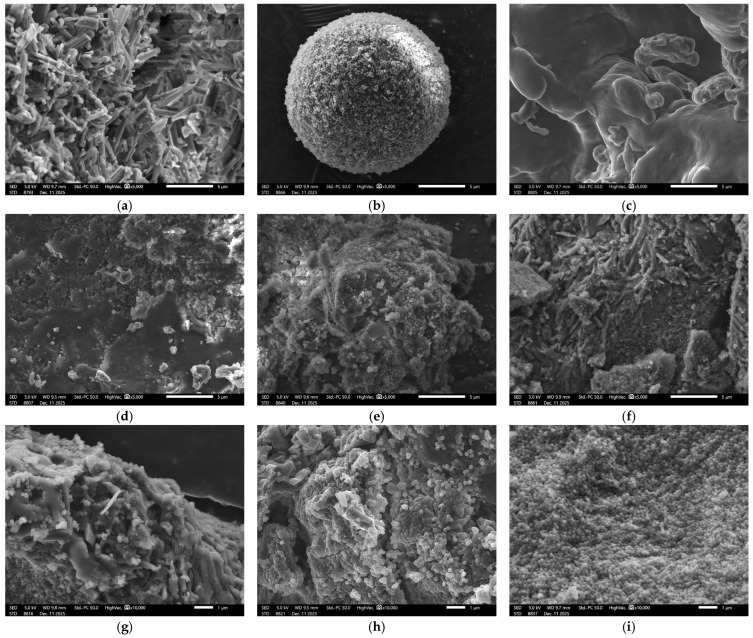
SEM micrographs of the surfaces of raw materials and pantoprazole formulations: (**a**) pantoprazole (5000×); (**b**) Eudragit^®^ S 100 (5000×); (**c**) HPMC (5000×); (**d**) F2 (5000×); (**e**) F9 (5000×); (**f**) F6 (5000×); (**g**) F2 (10,000×); (**h**) F6 (10,000×); (**i**) F9 (10,000×).

SEM revealed distinct morphological features for pantoprazole, the individual excipients, and the different matrix formulations. Pure pantoprazole exhibited an aggregated crystalline morphology composed of irregularly shaped crystals with heterogeneous size, which could be readily distinguished from the polymeric components (Figure 5a).

In the matrix mini-tablets, pantoprazole crystals appear to be progressively embedded within the polymeric network. A clear reduction in exposed drug crystals is observed as the proportion of HPMC is increased, indicating effective encapsulation of the API within the hydrophilic matrix. This morphological trend is consistent with the dissolution results, particularly for formulation F2 (Figure 5d,g), which shows minimal drug release during the first 2 h under acidic conditions, likely due to the enhanced matrix coverage and protection of pantoprazole.

SEM images of Eudragit^®^ S 100 recorded at the same magnification (5000×) show agglomerates of small, spherical particles (Figure 5b). In the formulations, these particles are observed on the surface of HPMC and pantoprazole, contributing to increased surface roughness. Due to their smaller particle size compared with HPMC, formulations containing higher proportions of Eudragit^®^ S 100 show regions where pantoprazole crystals remained partially uncovered. This effect was particularly evident in formulation F9 (Figure 5e,i), where exposed crystalline features of pantoprazole are clearly visible, consistent with the increased drug release observed during the gastric phase.

HPMC appears as a continuous, amorphous mass, reflecting the entangled nature of its polymer chains (Figure 5c). In formulations with higher HPMC content, larger compact masses with surface irregularities are observed, modulated by the relative proportion of Eudragit^®^ S 100. In these systems, pantoprazole crystals are scarcely detectable or absent from the surface. A representative example is formulation F2 (Figure 5d,g), where a dense, paste-like structure formed by compressed HPMC is uniformly covered with Eudragit^®^ S 100, with no visible pantoprazole crystals. Similar morphological characteristics are observed for formulation F6 (Figure 5f,h), supporting efficient drug embedding within the polymeric matrix.

Overall, the SEM analysis indicates that the spatial distribution and surface exposure of pantoprazole within the polymeric matrices strongly depend on the relative proportions of HPMC and Eudragit^®^ S 100. Formulations with a higher HPMC content exhibit a more homogeneous and compact matrix structure, effectively embedding the drug. These observations provide structural support for the dissolution behaviour, and reinforce the role of polymer selection and matrix architecture as key determinants of controlled drug-release performance.

### 3.6. Mini-Tablet Physical Properties

The physical properties of pantoprazole matrix mini-tablets containing HPMC or Eudragit^®^ S 100 are summarised in Table 5. The variability observed among formulations provides valuable insight into the internal structure of the matrices and their potential influence on drug-release mechanisms.

The mean tablet mass ranged from 124.2 ± 3.02 mg to 128.1 ± 4.14 mg and complied with the pharmacopoeial requirements for mass uniformity, as the percentage deviation did not exceed ±7.5%. The average weight of the mini-tablets showed excellent uniformity, with no significant differences identified between formulations. One-way ANOVA, Levene’s test (*p* = 0.703), confirmed homoscedasticity, while Tukey’s HSD did not reveal significant differences in weight, as shown in Table 5. Dimensional parameters (thickness and diameter) were determined according to the specifications of the RFE, and are also reported in Table 5. Among the formulations, F9 showed the highest friability, suggesting comparatively reduced mechanical robustness.

Friability values remained below the acceptance limit of 1%, ranging from 0.09 to 0.56%, indicating adequate mechanical resistance for handling, packaging, and transport. However, the relatively higher friability observed in F9 suggests a weaker underarticulate bonding in the matrix. This behaviour may be related to differences in polymer distribution or insufficient solid bridges between excipient particles.

While commercial pantoprazole tablets typically achieve high mechanical strength through a multilayer film coating, developed mini-tablet hardness (crushing strength) ranged from 76.5 ± 4.74 N to 96.95 ± 4.52 N—remarkably high for such a small diameter (4 mm) and when compared with other studies in the bibliography of [32]. This is explained by the high compactability of the HPMC/Eudragit^®^ S 100 blend. During the direct compression, HPMC acts as a dry binder that undergoes plastic deformation, creating strong interparticle bonds, while Eudragit^®^ S 100 fills the residual voids, as reflected in previous studies [31,32,33].

Despite the utilisation of analogous compression settings during the manufacturing process, substantial discrepancies were identified in the drug-release profiles [7]. Tablet hardness is a pivotal factor in determining the internal matrix structure. Higher hardness is generally associated with reduced porosity and increased tortuosity of the diffusion pathways. This may impede solvent penetration and retard drug release. In contrast, lower hardness is generally linked to a more porous structure, which enables faster fluid uptake and accelerates drug-release kinetics [34,35,36,37]. Importantly, the lack of direct correlation between compression parameters and drug-release behaviour highlights that matrix performance is governed by a complex interplay between mechanical properties, polymer characteristics, and matrix microstructure [6,8,31].

In this context, formulation F5 (≈97.0 N) exhibited the highest hardness, which may generate a more compact matrix, hinder solvent penetration, and reduce drug diffusion through the matrix. On the other hand, formulation F1 may allow for faster medium ingress and matrix hydration, leading to enhanced drug diffusion, as shown in prior studies [15,36]. Meanwhile, formulation F9 could indicate structural heterogeneity, which may result in irregular drug-release behaviour.

The physical and functional properties of the developed mini-tablets (F1–F9) were subjected to a rigorous statistical evaluation to confirm the significance of the observed differences. The homogeneity of variance was confirmed for tablet hardness using Levene’s test (F (8,18) = 1.07, *p* = 0.422), justifying the use of parametric one-way ANOVA. Most of the formulations (F1–F4 and F6–F8) showed no differences among them (*p* > 0.05), forming Group a, which represents the standard behaviour of HPMC / Eudragit S100. However, F5 and F9 were identified as statistically independent groups (Groups b and c, respectively). Subsequently, the statistical analysis of mini-tablet friability yielded Levene’s test F (8,18) = 0.337, *p* = 0.9399, and so the assumption of homogeneity of variances was confirmed. Formulations F8 and F9 differed significantly from several other groups (*p* < 0.05). In contrast, no significant differences were observed among formulations F1–F7 (*p* > 0.05).

Finally, from the disintegration results after two hours of the acid phase, formulations F4, F5, F7, and F8 were seen to be crashing in the first two hours. Acceptance criteria in this study included the absence of any signs of disintegration, cracking, or swelling. Subsequently, the rest of the formulation units were transferred to phosphate buffer pH = 6.8, where they showed complete releasefor up to 24 h at 37 °C.

### 3.7. Multivariate Statistical Analysis of Formulation Performance (PCA and K-Means Clustering)

**Figure 6:**
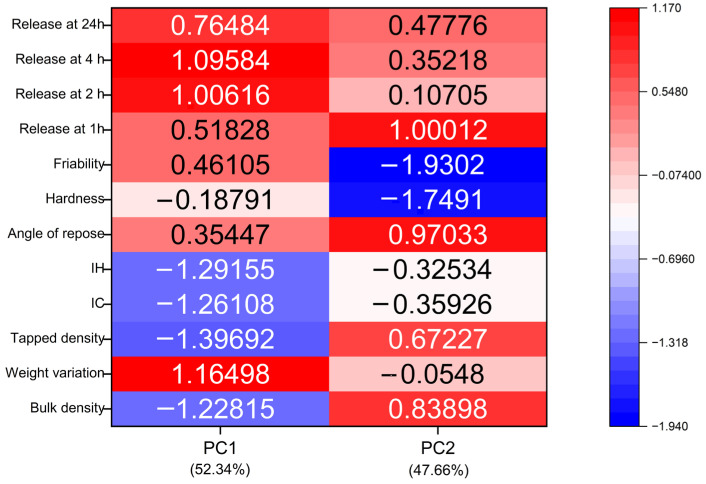
Heatmap of PCA loadings for the physico-chemical and biopharmaceutical variables. The colour scale represents the correlations between the variables (flow properties, tablet characteristics, and dissolution profiles) and the first two principal components (PC1 and PC2). Red indicates a strong positive correlation, whereas blue indicates a strong negative correlation.

Principal Component Analysis (PCA) proved to be a robust multivariate tool to discriminate among the developed formulations by establishing correlations between their physico-chemical attributes and drug release at 24 h (Figure 6). This analysis enabled the identification of the variables that contributed most to overall variability. Both components displayed significant eigenvalues of 6.125 and 2.96, respectively, significantly exceeding the Kaiser criterion (eigenvalue > 1) and confirming a strong discriminatory powder. The first principal component (PC1), representing 52.34% of the data, showed strong positive correlations with drug release at 1, 2, 4, and 24 h, as well as with the angle of repose and mean tablet weight, while displaying negative correlations with hardness, friability, and the compressibility indices (IH and IC). These relationships indicate that PC1 primarily differentiates formulations according to drug-release performance rather than mechanical strength. 

The second principal component (PC2), representing the 47.66% of the data, exhibited positive correlations with the bulk and tapped densities and a negative correlation with friability. Therefore, PC2 reflects the relationship between density-related packing/cohesion and structural fragility, as shown in Figure 6.

Overall, the PCA results suggest that formulations with higher drug release tend to exhibit lower mechanical resistance, whereas formulations with a higher density and compactness display improved structural robustness. In this regard, PC1 separates formulations with enhanced release capacity (positive scores) from those with greater mechanical strength (negative scores), while PC2 differentiates cohesive, well-compacted systems (positive scores) from less dense and more brittle formulations (negative scores).

To further explore the relationships among the parameters identified by PCA, a k-means clustering analysis was performed and visualised in a PCA biplot, enabling a straightforward interpretation of formulation grouping and variable interactions. Although PCA yielded three principal components, only PC1 and PC2 were retained for interpretation because together they explained 82% of the cumulative variance. The biplot displays the formulations (F) projected onto the PC1 and PC2 space according to their physical attributes and drug-release behaviour.

Formulations F3 and F9 exhibit negative PC1 scores, indicating a weaker association with drug release and a stronger influence of mechanical and packing-related features. These two formulations were clearly distinguished by their PC2 positioning. F3 shows positive PC2 values, consistent with higher bulk and tapped densities and improved packing and cohesion, whereas F9, located in the lower region with negative PC2 values, is associated with lower density and poorer compactability. This behaviour is consistent with its slow and incomplete release, not exceeding 41%.

Among formulations with positive PC1 scores (F1, F2, and F6), the modulating contribution of PC2 becomes particularly relevant. Although F1 and F6 show similar PC1 scores, differences along PC2 are associated with distinct release profiles, suggesting that PC2-related physico-chemical attributes become critical once PC1 conditions are met. Notably, F2 and F6, located in the intermediate region of the biplot, exhibit a balanced interaction between compactability and drug diffusion, resulting in a stable and sustained 24 h release. Overall, this pattern reflects optimal behaviour by combining adequate packing and cohesiveness (positive PC2) with improved release performance (positive PC1).

Following a comprehensive multivariate evaluation, F2 and F6 were identified as the most promising candidates, exhibiting an optimal balance between mechanical robustness and controlled drug diffusion, thereby meeting the stringent prolonged-release criteria established for the designated dosage form. This is in contrast to F3, which exhibited a less controlled release pattern, and F9, which demonstrated slow and insufficient overall release. Furthermore, formulations exhibiting anomalous transport kinetics (e.g., F6) suggest a favourable balance between water permeation and controlled polymer relaxation, supporting sustained delivery while maintaining matrix integrity over the 24 h period. This is of particular relevance for acid-labile APIs (e.g., pantoprazole), for which reliable post-gastric release is required.

**Figure 7:**
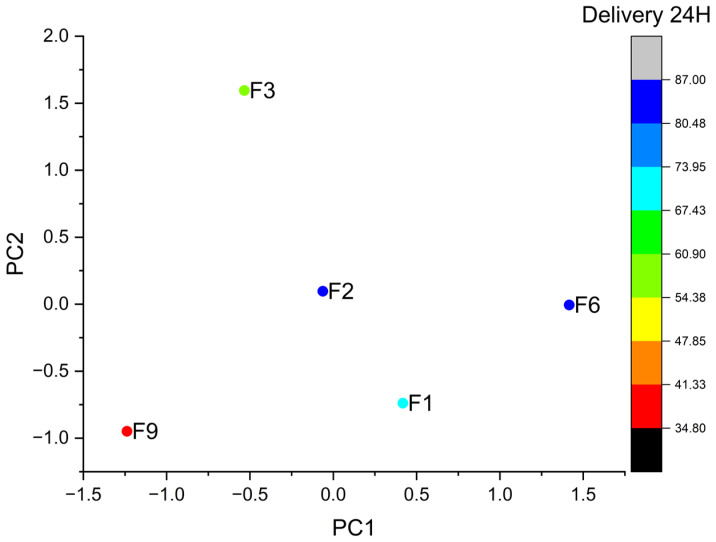
PCA score plot of the selected formulations. The distribution of formulations (F1 to F9) is shown along the first two components (PC1 and PC2). The colour scale represents drug release at 24 h, with blue indicating higher release and red indicating lower release.

These findings corroborate the kinetic analysis, indicating that the optimal formulations (F2 and F6) achieve controlled release through a balanced interplay between matrix integrity and polymer relaxation. In contrast, the more extreme profiles of F1 and F3 are predominantly diffusion-driven, whereas F9 appears to lack sufficient structural dynamics to support efficient anomalous transport, as shown in Figure 7.

### 3.8. Drug Dissolution Profiles and Mathematical Kinetic Modelling of Matrix-Based Formulations

**Figure 8:**
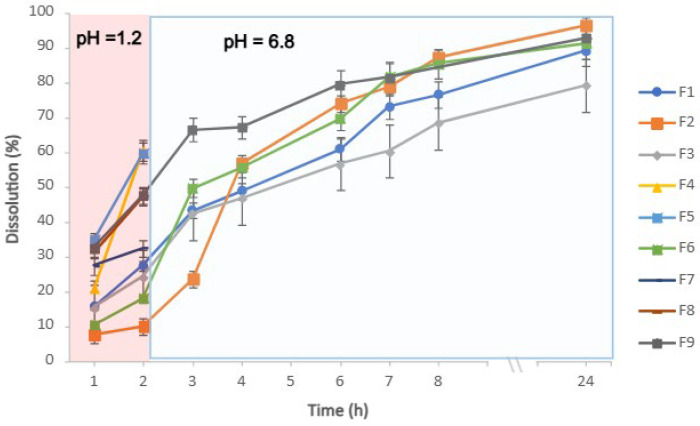
Dissolution profiles of pantoprazole formulations. First two hours in acidic medium (pH 1.2). Afterwards, the mini-tablets that suffered physical alterations (F4, F5, F7, and F8) were removed from the study. Tablets that remained physically intact (F1, F2, F3, F6, and F9) continued to be studied under phosphate buffer (pH 6.8) media at 37 °C during 22 h, for a total of 24 h.

The in vitro dissolution profile of pantoprazole matrix mini-tablets was evaluated using a USP dissolution apparatus in buffer media at different pH values (1.2–6.8). The test was initiated in 0.1 M HCl, and is shown in Figure 8. A critical requirement for the developed gastro-resistant systems was to maintain drug release below 10% during the first 2 h (acid stage). Accordingly, F2 was identified as the most robust formulation, followed closely by F6, which exhibited 7.2% release at 2 h. Therefore, F2 and F6 were selected as the most suitable candidates for sustained delivery. A key difference was observed in the dissolution profiles between formulation F2, predominantly composed of HPMC—a time-dependent polymer—which exhibited a slower release profile, resulting in a rightward shift of the dissolution curve. In contrast, F6, which contains a higher proportion of the pH-dependent polymer, showed a faster drug release after the transition to pH 6.8, leading to a steeper release slope, as illustrated in Figure 8.

In contrast, formulations F1, F3, F5, and F9 showed substantial drug release during the initial 2 h. Nevertheless, dissolution testing was continued up to 24 h for these systems to assess the release kinetics of the matrices at different polymer ratios. Finally, formulations F4, F5, F7, and F8 were excluded from further development due to excessive premature release at early time points, which did not meet the gastro-resistant objectives of the dosage form. These formulations contain high proportions of the pH-dependent polymer but only a limited amount of HPMC. As a result, a greater fraction of pantoprazole crystals remains insufficiently embedded within the matrix, which may reduce the system’s ability to sustain drug release over extended periods, as previously reported by B.wilson et al. [12]. In our system, Eudragit^®^ S 100 acts as a protective network that limits water penetration at pH 1.2, while HPMC modulates drug release after the transition to pH 6.8. This complementary mechanism enables a more controlled and balanced release profile compared with single-polymer systems, as illustrated in study [6].

The drug-release profiles were evaluated at two critical time points to assess the enteric behaviour and the sustained release capacity. With release at 2 h (acid stage), the ANOVA indicated that formulation F2 exhibited unique kinetic behaviour, being significantly different (*p* < 0.0001) from all the other tested groups. This distinctive profile, identified as an independent group in Tukey’s post hoc test, suggests that the specific ratios of HPMC/Eudragit^®^ S 100 can create a transitional release state, providing further evidence of the high tunability of the proposed delivery system. The dissolution rate analysis revealed high sensitivity of the drug-release profile to the matrix composition. Levene’s test (F (8,18) = 0.93; *p* = 0.516) confirmed that the variances were homogeneous, allowing a robust one-way ANOVA. The 24 h drug release provides functional diversity of the formulations. Levene’s test (F (8,6) = 0.22; *p* = 0.971) confirmed an excellent homogeneity of variance. Post hoc analysis identified a group with sustained release (F1 and F2) that maintained matrix integrity, and other homogeneous groups exhibited intermediate behaviour (F6 and F9).

At the selected detection wavelength (285 nm), the spectra of HPMC and Eudragit^®^ S 100 did not show relevant overlap under the conditions used, supporting the specificity of the HPLC-UV quantification for pantoprazole. In addition, no interfering peaks from excipients or degradation products were observed at the retention time of pantoprazole, confirming adequate method specificity for the dissolution sample analysis.

**Figure 9:**
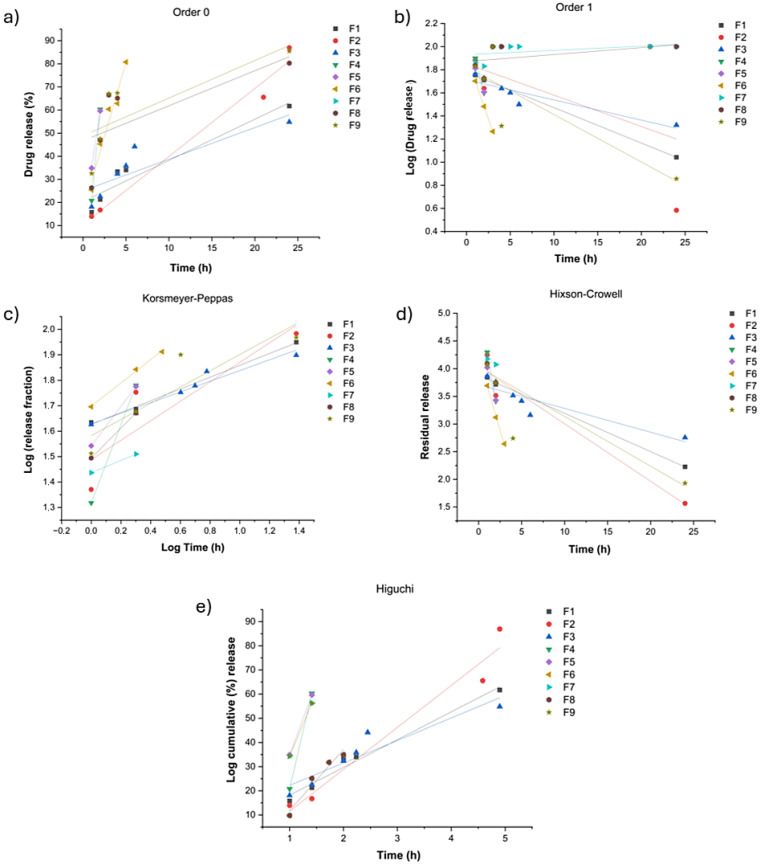
Linearised plots of the in vitro dissolution data for pantoprazole matrix mini-tablets (F1 to F9) fitted to common kinetic models: (**a**) Zero-order (cumulative drug release vs. time); (**b**) first-order (log drug release vs. time); (**c**) Korsmeyer–Peppas (log release fraction vs. log time); (**d**) Hixson–Crowell (residual release vs. time), and (**e**) Higuchi (log cumulative drug release vs. time). These representations were used to estimate the kinetic parameters and goodness-of-fit reported in Table 6.

The release data were fitted to several mathematical models (zero-order, first-order, Higuchi, Hixson–Crowell, and Korsmeyer–Peppas) to elucidate the predominant mass transport mechanism (Table 6 and Figure 9). The selection of best-fit model was based on (R^2^) and interpretation of the release exponent (n) from the Korsmeyer–Peppas powder law [30].

To facilitate mechanistic interpretation, the fitted models were considered according to their underlying assumptions.

Zero-order kinetics describes a release pattern in which the drug is released at an approximately constant rate independent of concentration, whereas first-order kinetics relates the release rate to the amount of drug remaining in the dosage form [10]. The Higuchi model assumes Fickian diffusion from a porous matrix and describes release as a function of the square root of time [6]. The semi-empirical Korsmeyer–Peppas model was used to describe more complex mechanisms and to estimate the release exponent (n) [10,11]. In general, n values of 0.45 indicate Fickian diffusion, values between 0.45 and 0.89 indicate anomalous (non-Fickian) transport involving both diffusion and polymer relaxation, and values above 0.89 are consistent with Case II transport dominated by polymer relaxation [10,11]. Finally, the Hixson–Crowell model relates release to changes in surface area and particle diameter due to progressive erosion [30]. Comparative assessment of model fitting allows for identification of the predominant release mechanism for each formulation, supporting the optimisation of matrix performance for sustained delivery, and, potentially, for reducing fluctuations in plasma drug exposure and improving adherence.

Overall, most formulations showed a better fit to the Korsmeyer–Peppas model (or related diffusion-based models). Formulations F4, F5, and F7 displayed an apparently excellent linearity (R^2^ = 1.00); however, both formulations rapidly disintegrated and reached a plateau after the first 2 h, indicating a loss of modified-release performance. Therefore, F4 and F5 were excluded from the kinetic interpretation.

Conversely, formulations F1, F3, and F6 showed a good fit to the Korsmeyer–Peppas model (R^2^ > 0.93), with release exponent (n) values of 0.43, 0.37, and 0.68, respectively. For F1 (n = 0.43) and F3 (n = 0.37), the results are consistent with Fickian diffusion-controlled release through the polymeric matrix, in agreement with Higuchi-type behaviour. Mechanistically, this suggests that the rate-limiting step is the drug’s outward diffusion through the aqueous channels of the swollen matrix. In these matrices, the HPMC hydrates to form a tortuous diffusion barrier; pantoprazole, being soluble at this pH, diffuses through the water-filled longitudinal channels of the gelled polymer. The fact n ≤ 0.45 confirms that the matrix structure remains relatively stable during the timeframe of the study, with negligible contribution from polymer relaxation or erosion [29,30].

In contrast, F6 (n = 0.68) exhibited anomalous (non-Fickian) transport, commonly observed in hydrophilic matrix systems, where drug release is governed by a combination of molecular diffusion and polymer relaxation/swelling, as reported by Barmpalexis et al. [30]. The hybrid composition is particularly effective because it prevents the quick-release characteristic of Fickian’s release by revealing the chain relaxation in Eudragit^®^ S 100 reaching its pK_a_, creating electrostatic repulsion that facilitates matrix expansion, while HPMC enables complete release through controlled matrix degradation [3]. This synergy, the contribution of diffusion and matrix hydration/erosion, results in more controlled release, governed by the gradual relaxation of the polymer chains.

Formulation F2 showed the best fit to the Hixson–Crowell model (R^2^ = 0.99), suggesting that drug release is influenced by changes in the surface area of the matrix as it dissolves and/or undergoes erosion. This kinetic behaviour is closely correlated with its compositions, specifically the presence of Eudragit^®^ S 100. At pH 6.8, the carboxylic groups of this enteric polymer undergo near complete ionisation, leading to gradual erosion [34,35,36]. Although the Higuchi model also showed good correlation (R^2^ = 0.96), it does not fully capture the contribution of surface-area reduction over time. In F2, the dissolution of the Eudragit^®^ S 100 dominates the release process and the higher proportion of HPMC (soluble in the release medium) may promote increases in viscosity, while decreases in polymeric chain mobility and relaxation might play a role in retardation of drug release, dimensional changes, reduce the wettability, supporting the applicability of Hixson–Crowell kinetics [6,31]. Overall, this finding suggests robust and reproducible performance in terms of matrix integrity and release behaviour.

For the remaining formulations, R^2^ values below 0.90 were obtained for most models, indicating that their release profiles are governed by more complex mechanisms that are not adequately described by the classical models evaluated.

### 3.9. Future Prospects

Future research should prioritise the in vivo validation of the optimised gastro-resistant mini-tablets to establish a robust in vitro–in vivo correlation (IVIVC). Additionally, exploring the formulations under biorelevant and dynamic gastrointestinal conditions, accounting for pH variability and transit scenarios, will be essential to confirm the reliability of the pH-shift performance. A more detailed mechanistic characterisation of the matrix, focusing on gel dynamics, swelling kinetics, and erosion-related mechanical integrity, is warranted to bridge the gap between microstructure and macro release behaviour. Furthermore, scale-up feasibility and manufacturing robustness must be addressed to ensure batch-to-batch reproducibility. Ultimately, the modular nature of this multiarticulate platform opens significant avenues for personalised medicine, including the development of tailored polypill concepts by combining units with distinct release profiles.

## 4. Conclusions

This study demonstrates the feasibility of developing gastro-resistant pantoprazole mini-tablets by a single-step direct-compression approach as an alternative to conventional enteric-coating strategies. Formulation performance was governed by a critical balance between mechanical integrity and drug-release behaviour. Under pH-shift dissolution conditions, the selected matrices met the gastro-resistant criterion during the acidic stage and provided sustained release up to 24 h, with release kinetics indicating combined diffusion- and erosion-related contributions. Solid-state and morphological analyses supported these findings by showing reduced apparent drug crystallinity and effective embedding of pantoprazole within the polymeric matrices.

Multivariate analysis (PCA and clustering) proved valuable for rational formulation optimisation by identifying the key variables driving performance and discriminating formulations according to release capacity versus mechanical robustness. Overall, these results support a simplified, coating-free manufacturing strategy that may reduce processing steps, avoid organic solvents, and lower production costs while maintaining controlled-release functionality.

## Figures and Tables

**Table 1:** Composition of mini-tablet formulations by direct-compression method using Eudragit^®^ S100 and HPMC.

Formulations	Composition (% *w*/*w*)			
	Pantoprazole	HPMC	Eudragit^®^ S 100	Compritol^®^ 888 ATO
F1	16	65	15	4
F2	16	60	20	4
F3	16	55	25	4
F4	16	50	30	4
F5	16	45	35	4
F6	16	40	40	4
F7	16	30	50	4
F8	16	20	60	4
F9	16	15	65	4

**Table 2:** Summary of kinetic models used for dissolution data fitting (equations and linearised forms).

Kinetics Model	Main Equation	Linear Graphical Representation	Refs.
Zero-Order	Qt = k0⋅t	Qt vs. t	[27,28,29]
First-Order	log(Qr) = log(Q0) − 2.303k1⋅t	log(Qr) vs. t	[29]
Higuchi	Qt = kH⋅t	Qt vs. t	[5,29]
Hixson–Crowell	$Q_{0}^{1/3}-Q_{T}^{\frac{1}{3}}=k_{HC}\;t$	3Qr vs. t	[30]
Korsmeyer–Peppas	$\frac{M_{t}}{M_{\infty }}={kt}^{n}$	log(M∞Mt) vs. log(t)	[12,30]

**Table 3:** Physical and flowability parameters of the powder blends (F1–F9).

Formulation	Angle of Repose θ	Bulk Density (g/mL)	Tapped Density (g/mL)	Carr’s Index (%)	Hausner’s Ratio
F1	17.62 ± 0.07	0.48 ± 0.05	0.535 ± 0.01	9.52 ± 0.15	1.10 ±0.05
F2	24.56 ± 0.09	0.45 ± 0.03	0.568 ± 0.04	20.00 ± 0.14	1.25 ± 0.03
F3	20.56 ± 0.10	0.73 ± 0.09	0.97 ± 0.06	24.39 ± 0.14	1.32 ± 0.03
F4	20.03 ± 0.07	0.45 ± 0.03	0.55 ± 0.07	18.40 ± 0.11	1.22 ± 0.03
F5	21,91 ± 0.19	0.45 ± 0.04	0.50 ± 0.02	11.11 ± 0.13	1.125 ± 0.01
F6	26.97 ± 0.08	0.48 + 0.04	0.55 ± 0.02	11.36 ± 0.15	1.13 ± 0.06
F7	16.02 ± 0.08	0.43 ± 0.01	0.49 ± 0.05	12.00 ± 0.15	1.13 ± 0.04
F8	17.56 ± 0.05	0.36 + 0.02	0.47 ± 0.02	23.81 ± 0.12	1.31 ± 0.01
F9	13.30 ± 0.06	0.44 ± 0.05	0.55 ± 0.09	21.15 ± 0.13	1.26 ± 0.05

**Table 4:** DSC thermal parameters of the raw materials: temperatures (T_onset_, T_peak_) and enthalpy of fusion (∆H_fusion_).

Samples	T_onset (fusion)_/C°	T_peak(fusion)_/C°	∆H_fusion_/Jg^−1^	Reported Melting Point
Pantoprazole	136.22	140.49	88.99	[25]
HPMC	125.79	119.79	66.75	[26]
Eudragit^®^ S 100	161.35	162.72	47.28	[1]
Compritol^®^ 888 ATO	69.98	75.86	115.51	[24]

**Table 5:** Physical properties of specific direct compression.

Formulation	Hardness (kg/cm^2^) ± SD	Friability (%)	Weight Variation (mg) ± SD	Thickness ± SD	Diameter ± SD
F1	82.8 ± 5.6 ^a^	0.17 ± 0.09 ^a^	128.1 ± 4.14 ^a^	6.026 ± 0.010	4.27 ± 0.110
F2	87.08 ± 6.2 ^a^	0.31 ± 0.08 ^ab^	126.15 ± 5.19 ^a^	6.022 ± 0.038	4.27 ± 0.110
F3	85.35 ± 8.02 ^a^	0.16 ± 0.10 ^bc^	126.65 ± 3.21 ^a^	6.028 ± 0.037	4.26 ± 0.085
F4	84.57 ± 7.95 ^a^	0.09 ± 0.11 ^c^	127.1 ± 3.85 ^a^	5.99 ± 0.009	4.14 ± 0.150
F5	96.95 ± 4.52 ^b^	0.22 ± 0.07 ^c^	124.2 ± 3.02 ^a^	5.99 ± 0.017	4.13 ± 0.144
F6	76.57 ± 6.58 ^a^	0.15 ± 0.08 ^c^	127.2 ± 2.78 ^a^	6.05 ± 0.007	4.16 ± 0.133
F7	87.25 ± 8.09 ^a^	0.15 ± 0.09 ^c^	124.3 ± 3.31 ^a^	6.00 ± 0.013	4.14 ± 0.020
F8	76.88 ± 8.61 ^a^	0.56 ± 0.12 ^c^	124.45± 2.85 ^a^	6.00 ± 0.002	4.17 ± 0.13
F9	94.46 ± 10.17 ^c^	0.66 ± 0.14 ^c^	126.05 ± 3.79 ^a^	6.00 ± 0.007	4.15 ± 0.013

Physical characterisation of the tablets: Weight, hardness, and friability. Data are expressed as mean ± SD (n = 3). Values within the same column with different superscript letters indicate statistically significant differences (one-way ANOVA, Tukey’s post hoc).

**Table 6:** Kinetic-model parameters (k and n) and goodness-of-fit (R^2^) derived from pH-shift dissolution profiles of mini-tablet formulations prepared by direct compression.

Formulation	Zero-Order Kinetics		First-Order Kinetics		Higuchi		Hixson–Crowell		Korsmeyer–Peppas	
	K1	R^2^	K1	R^2^	Kh	R^2^	Khc	R^2^	n	R^2^
F1	1.78	0.91	0.01	0.99	11.49	0.98	0.04	0.99	0.43	1.00
F2	2.94	0.92	0.03	0.88	17.36	0.96	0.09	0.99	0.6	0.98
F3	1.36	0.73	0.01	0.80	9.29	0.87	0.03	0.78	0.37	0.93
F4	39.54	-	0.23	-	95.38	-	0.88	-	1.53	-
F5	24.80	-	0.21	-	59.87	-	0.59	-	0.78	-
F6	12.82	0.95	0.13	0.94	39.13	0.95	0.30	0.93	0.68	0.97
F7	21.86	-	0.01	-	12.94	-	0.09	-	0.77	-
F8	1.51	0.5	0.07	0.86	10.57	0.62	0.05	0.61	0.31	0.70
F9	1.62	0.59	0.02	0.81	11.1	0.71	0.05	0.74	0.28	0.78

## Data Availability

The original contributions presented in this study are included in the article. Further inquiries can be directed to the corresponding author.

## References

[1] Blynskaya E.V., Tishkov S.V., Vinogradov V.P., Alekseev K.V., Marakhova A.I., Vetcher A.A. (2022). Polymeric Excipients in the Technology of Floating Drug Delivery Systems. Pharmaceutics.

[2] Grund J., Koerber M., Walther M., Bodmeier R. (2014). The effect of polymer properties on direct compression and drug release from water-insoluble controlled release matrix tablets. Int. J. Pharm..

[3] Vemula S.K. (2015). A Novel Approach to Flurbiprofen Pulsatile Colonic Release: Formulation and Pharmacokinetics of Double-Compression-Coated Mini-Tablets. AAPS PharmSciTech.

[4] Varma M.V.S., Kaushal A.M., Garg A., Garg S. (2004). Factors affecting mechanism and kinetics of drug release from matrix-based oral controlled drug delivery systems. Am. J. Drug Deliv..

[5] Mondal N. (2018). The role of matrix tablet in drug delivery system. Int. J. Appl. Pharm..

[6] Ólafsdóttir S.Á., Magnúsdóttir A.V., Geirsdóttir J.B., Sigurðardóttir M.S. (2025). Development of Caffeine Tablets with Dual Burst Release. Bachelor’s Thesis.

[7] Hirun N., Kraisit P. (2022). Drug-polymers composite matrix tablets: Effect of hydroxypropyl methylcellulose (hpmc) k-series on porosity, compatibility, and release behavior of the tablet containing a BCS Class I drug. Polymers.

[8] Trisopon K., Saokham P., Kittipongpatana N., Chomchoei N., Kittipongpatana O.S. (2025). Synergistic co-processing of heat-moisture treated resistant rice starch with HPMC and Eudragit® S100: A novel multifunctional excipient for direct compression and colon-targeted delivery. Eur. J. Pharm. Biopharm..

[9] Veerareddy P.R., Vemula S.K. (2012). Formulation, evaluation and pharmacokinetics of colon targeted pulsatile system of flurbiprofen. J. Drug Target..

[10] Obeidat W.M., Nokhodchi A., Alkhatib H. (2015). Evaluation of matrix tablets based on Eudragit®E100/Carbopol®971P combinations for controlled release and improved compaction properties of water soluble model drug paracetamol. AAPS PharmSciTech.

[11] Jayantilal K., Baliram R., Janrao M. (2012). Formulation and evaluation of sustained release tablets of metformin hydrochloride by solid dispersion technique using ph dependent and ph independent Eudragit Polymers. Ars Pharm..

[12] Wilson B., Babubhai P.P., Sajeev M.S., Jenita J.L., Priyadarshini S.R.B. (2013). Sustained release enteric coated tablets of pantoprazole: Formulation, in vitro and in vivo evaluation. Acta Pharm..

[13] Mamani P.L., Ruiz-Caro R., Veiga M.D. (2012). Matrix Tablets: The effect of hydroxypropyl methylcellulose/anhydrous dibasic calcium phosphate ratio on the release rate of a water-soluble drug through the gastrointestinal tract I. In vitro Tests. AAPS PharmSciTech.

[14] Patra C.N., Priya R., Swain S., Jena G.K., Panigrahi K.C., Ghose D. (2017). Pharmaceutical significance of Eudragit: A review. Futur. J. Pharm. Sci..

[15] Moussa E., Siepmann F., Flament M., Benzine Y., Penz F., Siepmann J., Karrout Y. (2019). Controlled release tablets based on HPMC:lactose blends. J. Drug Deliv. Sci. Technol..

[16] Verma A., Dubey J., Hegde R.R., Rastogi V., Pandit J.K. (2016). Helicobacter pylori: Past, current and future treatment strategies with gastroretentive drug delivery systems. J. Drug Target..

[17] Step, “ICH Guideline Q9 (R1) on Quality Risk Management.” [Online]. https://www.ema.europa.eu/en/ich-q9-quality-risk-management-scientific-guideline.

[18] Real Academia de Medicina (2023). 2.9.34. Densidad aparente y densidad después de asentamiento de los polvos. Real Farmacopea Española.

[19] Real Academia de Medicina (2023). 2.9.36. Flujo de polvo. Real Farmacopea Española.

[20] Real Academia de Medicina (2023). 2.9.5 Uniformidad de masa de las preparaciones presentadas en dosis únicas. Real Farmacopea Española.

[21] Real Academia de Medicina (2023). 2.9.7. Friabilidad de los comprimidos no recubiertos. Real Farmacopea Española.

[22] Real Academia de Medicina (2023). 2.9.1. Disgregación de comprimidos y cápsulas. Real Farmacopea Española.

[23] Nokhodchi A., Raja S., Patel P., Asare-Addo K. (2012). The role of oral controlled release matrix tablets in drug delivery systems. BioImpacts.

[24] Real Academia de Medicina (2023). 2.9.6. Uniformidad de contenido de las preparaciones unidosis. Real Farmacopea Española.

[25] Step. Committee for Human Medicinal Products ICH Guideline Q8 (R2) on Pharmaceutical Development. 2017. [Online]. https://www.ema.europa.eu/en/ich-q8-r2-pharmaceutical-development-scientific-guideline.

[26] Real Academia de Medicina (2023). 2.9.3. Ensayo de disolución de las formas farmacéuticas sólidas. Real Farmacopea Española.

[27] Ahmed T.A., Suhail M.A.A., Hosny K.M., Abd-Allah F.I. (2017). Clinical pharmacokinetic study for the effect of glimepiride matrix tablets developed by quality by design concept. Drug Dev. Ind. Pharm..

[28] Mujtaba A., Kohli K. (2016). In vitro/in vivo evaluation of HPMC/alginate based extended-release matrix tablets of cefpodoxime proxetil. Int. J. Biol. Macromol..

[29] Karvekar M., Khan A.B. (2017). A Brief Review on Sustained Release Matrix Type Drug Delivery System. J. Pharm. Res..

[30] Cho J., Kim D., Yi J.S., Park S. (2021). Microarchitecture of polyvinylidene fluoride-bound self-standing microporous layer and its implication to water management in fuel cells. J. Power Sources.

[31] Malik F.D., Singh I. (2012). Formulation and evaluation of press coated tablets of esomeprazole for colonic delivery. Asian J. Pharm..

[32] Hadi M.A., Rao N.G.R., Rao A.S. (2014). Formulation and evaluation of pH-responsive mini-tablets for ileo-colonic targeted drug delivery. Trop. J. Pharm. Res..

[33] Piao Z.Z., Lee K.-H., Kim D.-J., Lee H.-G., Lee J., Oh K.T., Lee B.-J. (2010). Comparison of release-controlling efficiency of polymeric coating materials using matrix-type casted films and diffusion-controlled coated tablet. AAPS PharmSciTech.

[34] Barmpalexis P., Kachrimanis K., Malamataris S. (2018). Statistical moments in modelling of swelling, erosion and drug release of hydrophilic matrix-tablets. Int. J. Pharm..

[35] Mustafa W.W., Fletcher J., Khoder M., Alany R.G. (2022). Solid Dispersions of Gefitinib Prepared by Spray Drying with Improved Mucoadhesive and Drug Dissolution Properties. AAPS PharmSciTech.

[36] Sonar G.S., Rawat S. (2015). Formulation and design of Multiunit particulate system (MUPS) tablet of Pantoprazole by QbD: Effect of compression variables on the finished product. J. Appl. Pharm. Sci..

[37] El Gamal S.S., Naggar V.F., Allam A.N. (2011). Optimization of acyclovir oral tablets based on gastroretention technology: Factorial design analysis and physicochemical characterization studies. Drug Dev. Ind. Pharm..

